# Study of the Degree of Cure through Thermal Analysis and Raman Spectroscopy in Composite-Forming Processes

**DOI:** 10.3390/ma12233991

**Published:** 2019-12-02

**Authors:** Juan A. García-Manrique, Bernabé Marí, Amparo Ribes-Greus, Llúcia Monreal, Roberto Teruel, Llanos Gascón, Juan A. Sans, Julia Marí-Guaita

**Affiliations:** Institute of Design for Manufacturing and Automated Production, Universitat Politècnica de València (UPV), Camí de Vera s/n, 46022 València, Spain; bmari@fis.upv.es (B.M.); aribes@ter.upv.es (A.R.-G.); lmonreal@mat.upv.es (L.M.); r.teruel@upvnet.upv.es (R.T.); llgascon@mat.upv.es (L.G.); juasant2@upvnet.upv.es (J.A.S.); juliasetze@gmail.com (J.M.-G.)

**Keywords:** prepeg, carbon fiber, Raman spectroscopy

## Abstract

The curing of composite materials is one of the parameters that most affects their mechanical behavior. The inspection methods used do not always allow a correct characterization of the curing state of the thermosetting resins. In this work, Raman spectroscopy technology is used for measuring the degree of cure. The results are compared with conventional thermal gravimetric analysis (TGA), differential scanning calorimetry (DSC), and scanning electron microscope (SEM). Carbon fiber specimens manufactured with technologies out of autoclave (OoA) have been used, with an epoxy system Prepreg System, SE 84LV. The results obtained with Raman technology show that it is possible to verify the degree of polymerization, and the information is complementary from classical thermal characterization techniques such as TGA and DSC; thus, it is possible to have greater control in curing and improving the quality of the manufactured parts.

## 1. Introduction

There is currently a great demand from the aerospace, wind, nautical and automobile industries for the development of new high-performance composites. The cure characterization of the parts affects both the forming processes and the repair–maintenance processes. Cure time is generally calculated very conservatively to ensure complete curing of the part before removing it from the mold. However, this practice greatly slows manufacturing times, and in some cases, it damages the part. The “in situ” control techniques of the mold filling and cure monitoring provide enough information to reduce the injection and curing times. Biomedical applications are used to increase the thickness of the part’s fillers; however, a high degree of cure could be difficult to achieve, reducing its mechanical properties and the biocompatibility [1].

**Figure 1:**
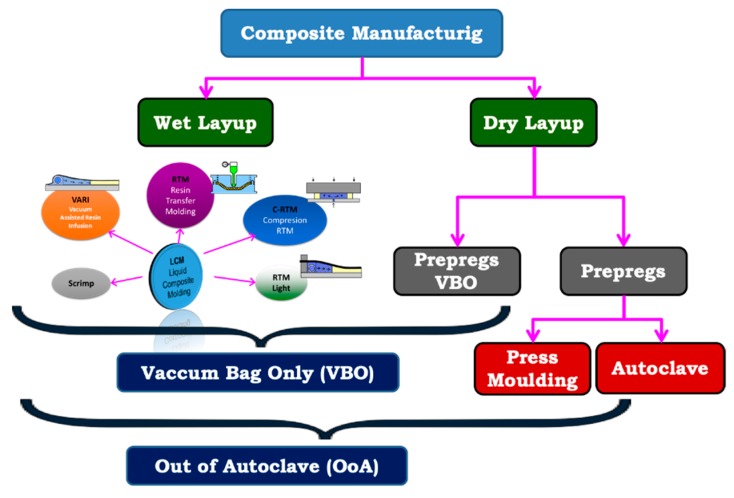
General classification of composite manufacturing process.

The manufacturing processes of composite materials can be divided into two large families: the wet layup and the dry layup processes (see Figure 1). Wet processes are those where the resin and the fabric have been mixed and fabrics already impregnated with the resin are supplied. The chemical polymerization reaction is “frozen” by the low supply temperature (−20 °C) and its reaction will be activated by heat. These materials are formulated with an excess of resin that must be evacuated during the fiber-compaction phase. When the resin is heated, its viscosity decreases and can flow through the thickness of the part. Thanks to this resin displacement through the thickness, the layers that form the laminate will be joined together. Autoclave, mechanical press, or atmospheric pressure can be used for compaction. This paper analyzes the forming processes compacted only with atmospheric pressure, which are known as VBO (vacuum bag only) or OoA (out of autoclave). These processes are those that have an easier processing; however, sometimes they are very expensive due to the large amount of labor they need, the cost of raw materials, etc.

On the other hand, regarding the dry layup forming processes, the impregnation phase of the fabrics takes place inside the mold. Therefore, the manufacturing process has a filling phase, a melt front advance, injection points, vents, etc. Depending on the type of injection or the stiffness of the countermold, it will define the different forming processes that make up the family of Liquid Composite Molding (LCM). These processes have great advantages over wet processes because they allow manufacturing with the same quality but at a significantly lower cost. Depending on the resin used, they may need an oven curing stage where the resin cures. However, these require a greater knowledge regarding the behavior of materials and manufacturing techniques than wet processes.

The objective of any composite manufacturing process is to impregnate the fabric with resin in the most efficient way and evacuate all the pores or voids that reduce the mechanical properties of the parts. In this context, the simulation of the formation and displacement of the pores has been an important research topic in recent years. An extensive review on this topic can be found in (Pillai, 2004 [2]; Park and Lee, 2011 [3]). Autoclave curing processes (OoA, out of autoclave) and vacuum bag-only compression (VBO, vacuum bag only) processes are often considered equivalent, but there are some differences between them. OoA processes, as the name implies, are all processes that heal without using an autoclave, but it is possible to apply a compaction pressure as large as necessary, for example, using a hot press for curing. However, concerning compaction processes with vacuum bag only, the compaction pressure is limited to atmospheric pressure. In both cases, the cost reduction with respect to autoclave manufacturing is important, and production times can be greatly reduced. In both cases, the manufacture of components out of autoclave with the same quality as those obtained with the autoclave is a major technological challenge. In this context, it is necessary to know the behavior of the materials during the forming process, and numerical models adapted to the different material scales are needed. In all forming processes, either wet (prepregs) or dry (LCM), numerical methods that include capillary effects and/or resin–air interface modeling should be used. The most advanced models in these techniques are based on numerical schemes by biphasic finite elements [4] that use the Stokes or Darcy’s flows models adapted to the biphasic behavior.

Nowadays, these models cannot solve the full geometry of the part on a macro scale, so reduced schemes are used for its numerical calculation. The equations to be solved are very similar for dry path processes where the flow to be studied is in-plane XY; as for the wet path, processes where the flow to be studied is basically through the thickness Z. The most traditional manufacturing process of high-performance composite parts is prepregs. These materials are made of fiber and resin and are supplied in sheets. To achieve the maximum mechanical properties, the laminate must undergo a compaction phase and a heat phase for resin curing. The material compaction is necessary to achieve the maximum fiber volumetric fraction, and in addition, the excess of resin with which the prepregs are manufactured must be evacuated. This excess of resin flows in through the thickness direction and is necessary to get the bond between each layer of the laminate. Maximum compaction is achieved using expensive autoclaves that are only accessible to certain high value-added industry such as aerospace or racing vehicles.

**Figure 2:**
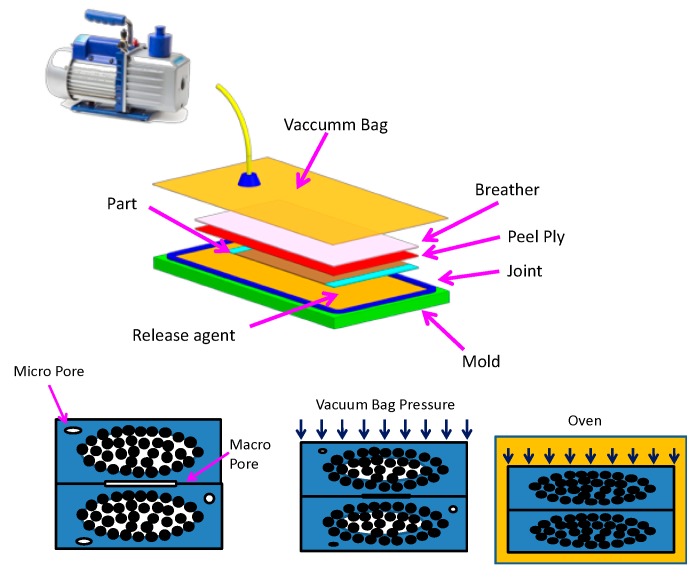
Prepreg technology.

The prepreg forming process (OoA) basically consists of three stages: layup, vacuum closing, and curing. First, the prepregs are cut in the required number of layers and sizes to obtain the thickness and shape of the part. Preimpregnated layers are stacked manually or using automated techniques (automatic tape layup). Intermediate compaction steps are performed to improve the layers compaction by applying vacuum at room temperature (see Figure 2). Once the part has reached the required thickness, it is compressed under vacuum, and the curing cycle begins. During the compaction phase, physical phenomena occur that are normally neglected. These phenomena include capillarity, drainage, inhibition, or pore mobility [5].

Therefore, the resin flows through the fabric and fills both the spaces between the tows and inside of the tows. The distances or gaps between the tows are around tenths of a millimeter, while the spaces inside the tows are 10–15 μm. Then, the problem needs a double-scale approximation, including the meso and the micro scale. Many authors have tried to solve this problem by modifying the permeability [6] and the subsequent treatment of flow equations as a traditional LCM problem. There is a difference of several magnitudes of order between the value of the permeability inside the tows (micro scale) and the space between the tows (macro or meso scale). As a result, the numerical simulation of the double-scale process is extremely complex. There are simplified models that estimate the saturation of the fabrics, but the work done so far does not consider many of the physical phenomena that occur inside the fabrics, resulting in them being inadequate for most applications of industrial interest. In this sense, it is worth highlighting the work carried out by Professor Veronic Michaud of the Ecole Polytechnique de Lausanne [7], who proposed a numerical model for the behavior of air permeability during the curing phase. Other researchers proposed the phenomenological characterization of the process, such as the research group of Professor S.G. Advani at the University of Delaware (USA) [8]. This approach requires the experimental data to have quantitative and qualitative results [9].

Composite manufacturers in the aerospace industry and other large industries, such as the automobile and wind industries, are looking for manufacturing processes out of autoclave (OoA) that can achieve 2% content in pores with less expensive and more efficient equipment. Autoclaves are used when the highest quality is needed in the final part, with a pore content of less than 2% and high glass transition temperatures (Tg). Aerospace autoclaves normally operate at 120 to 230 degrees Celsius within a nitrogen atmosphere at 7 bar pressure. It has been shown that processing with a preimpregnated vacuum bag only produces high porosity composite laminates, due to the air and moisture trapped in layers and between layers, which cannot be evacuated, and the lower (atmospheric) pressure cannot sufficiently compact. Moisture in prepreg can cause pores when processed only with the vacuum bag, but when processed in an autoclave, the higher pressure causes moisture to condense, suppressing pore growth. The first preimpregnated OoA was designed in the early 1990s for initial curing at low temperature (approximately 60 °C), followed by post-curing at high temperature (approximately 110 °C) [10].

The thermosetting resins used in the manufacture of high-performance composite (HPC) are initially processed in a liquid state, and by an exothermic curing reaction the polymer chains are crosslinked, forming covalent bonds of high hardness and chemical stability. When these properties are mixed with the appropriate fiber, they become an excellent composite material. The composite materials analyzed in this work are composed of a high modulus epoxy resin and long carbon fiber (HPC). The curing of this type of resin can be carried out by chemical methods, thermal methods, or a combination of both. The most commonly used method is the last one, since it allows greater control over the quality of the curing and reduces the manufacturing time. In all curing processes, not only the time and temperatures, but also the reaction rate must be controlled. There is an optimum in terms of cure temperature, which should coincide with the time when the reaction rate is higher. A higher temperature causes degradation of the material, rather than an increase in the reaction rate. The classic methods of resin characterization are differential scanning calorimetry (DSC) and dynamic mechanical analysis (DMA). These methods provide excellent information on the glass transition temperature, the heat generated by the chemical reaction, the cure rate, and the degree of cure. However, all these methods have the disadvantage of being destructive methods, and therefore can only be applied on a laboratory scale.

The cure reaction as a function of the manufacturing variables is a challenge for the analysis and design of processing operations. In addition, the physical properties of composite materials strongly depend on their microstructure and are directly related with the failure of the fibers or delaminations. Calorimetry will be used for the macrokinetic analysis of cure reactions. The cure reaction of epoxy resin will be analyzed also by means of thermal characterization using differential scanning calorimetry (DSC) and thermogravimetry (TGA). The vibrational properties of the composites were studied by Raman spectroscopy. The degree of cure of three samples of composite material with different levels of cure has been measured. The Raman spectroscopy vibrational method has been used and compared with those from thermal gravimetric analysis (TGA) and differential scanning calorimetry (DSC). It has been observed that complementary information can be obtained from classical trials, which allows progress toward non-destructive quality control in CFRP composites.

## 2. Materials and Methods

The coupons manufacturing begins with the consolidation stage. At this stage, the trapped air between the prepreg sheets is removed by vacuum sealing with a bag. This vacuum is applied for approximately 15 min at room temperature. Due to the stiffness of the prepreg layers, each of these consolidations are applied to 3–5 layers, depending on the total thickness of the part. In any case, the first layer is consolidated individually to ensure a good surface finish.

The SE 84LV is used for the manufacture of the coupons and can be cured at low temperature (85 °C) or, for faster molding of components, at 120 °C. Once the entire compaction sequence has been carried out, the mold is introduced into the oven (in-house manufacture) where the viscosity is reduced. The compaction pressure generates the flow of the resin, which mainly flows through the thickness of the part, and the resin excess is trapped in the absorption blanket. The polymerization reaction is activated by heat, and the oven must be programmed to control the resin reaction rate. The oven must control the temperature to avoid significant gradients inside the mold; therefore, the temperature rise ramp must be slow (approximately 1 degree/min). The oven temperature should be held for 10 h at 85 °C, and the tolerance must not exceed ±5 °C. Once the part is totally cured, quality control is recommended to ensure that no air has been trapped inside the part; the methods used are ultrasonic, stereography, thermography, etc.

A thermogravimetric study (TGA) of the samples has been carried out in order to simulate and optimize the percentage of volumetric fraction for the resin, fiber, and residue. Once the process conditions have been determined, the samples are introduced into a muffle furnace and the non-isothermal thermogravimetric multi-rate experiments are performed. Mettler-Toledo TGA/SDTA 851 (Mettler-Toledo, Columbus, OH, USA) equipment has been used. The samples weigh 10–11 mg and are heated in an alumina holder with a capacity of 70 μL. A heating curve from 30 °C to 1000 °C has been programmed with a rate of 10°/min in a controlled flow of oxygen atmosphere (50 mL/min) to simulate the real manufacturing conditions. Each experiment has been repeated three times, and finally, the characterization was carried out with the software STAR^®^ 9.10 from Mettler-Toledo. In accordance with ASTM D3171-11 [4], “Standard methods for constituent content of composite materials”, the weight percentage of each constituent is obtained from the following expressions:

(1) $$P1\left(\%\right)=\frac{P_{1}}{P_{0}}\cdot 100$$

(2) $$P2\left(\%\right)=\frac{P_{2}}{P_{0}}\cdot 100$$

(3) $$P3\left(\%\right)=\frac{P_{3}}{P_{0}}\cdot 100$$

(4) Residue (%) = P1 − P2 − P3

P_0_ = initial mass (5)

P_1_ = final mass (6)

P_3_ = carbon mass (7)

P_4_ = resin mass (8)

The degree of resin cure of the specimens has been measured by differential scanning calorimetry for later comparison with the results obtained from Raman spectroscopy. A Mettler Toledo DSC 822 DSC (Mettler-Toledo, Columbus, OH, USA) with samples of 4–6 mg weight was used and sealed in aluminium pans with a capacity for 40 μL. The programmed heating curve was from 25 to 300 °C with a heating rate of 10 °C/min for dynamic DSC scanning. The total heat of the reaction, HT, is obtained from the total area in the heat generation versus the time line graph. The degree of cure and the rate of the degree of cure will be determined in the isothermal scanning experiment. The measurements were made at temperatures from 125 to 145 °C with increments of 5 °C and a rate of heating of 10 degrees/min.

The isothermal curing curve is calculated from the total area enclosed by these exothermic curves. The samples are cooled and heated again (from 25 to 300 °C at 10 °C/min) after each isothermal scan to obtain the residual heat of the reaction, RH. This value is obtained from the area under the exothermic peak in the resulting curve. To carry out Raman spectroscopy tests, a backscattering geometry by a confocal HORIBA Jobin-Yvon LabRam high-resolution micro Raman spectrometer (HORIBA Jobin-Yvon, NJ, USA) has been used. The test characteristics were 1200 grooves/mm grating at a 100-μm slit and 50× objective, in combination with a thermoelectrically cooled multichannel CCD (charge-coupled device) detector (spectral resolution below 3 cm^−1^). A solid-state laser with a power of 50 mW emitting at 532.12 nm has been used.

## 3. Results and Discussion

**Figure 3:**
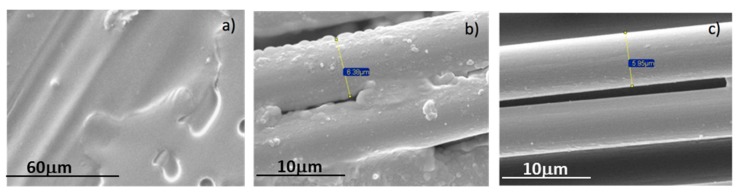
FESEM micrographs of the samples with different degree of cure: (**a**) Non-cured, (**b**) Semi-cured, (**c**) Cured.

The degree of cure of three composite samples with different characteristics has been analyzed. Sample 1 is a non-cured sample, which is used as the reference. It is a carbon prepreg sheet with any degree of cure, corresponding to a sample at the beginning of the manufacturing process. Sample 2 has been semi-cured out of autoclave, and Sample 3 was completely cured also in OoA. Figure 3 shows the FESEM micrographs for the three studied samples with different degrees of cure. The effect of the cure time is evident in these micrographs, the resins covering the carbon fibers disappear with the evolution of the cure reaction. According to Figure 3, the diameter of carbon fibers was found to be about 6 µm.

### 3.1. Derivative Thermogravimetric (DTG)

**Figure 4:**
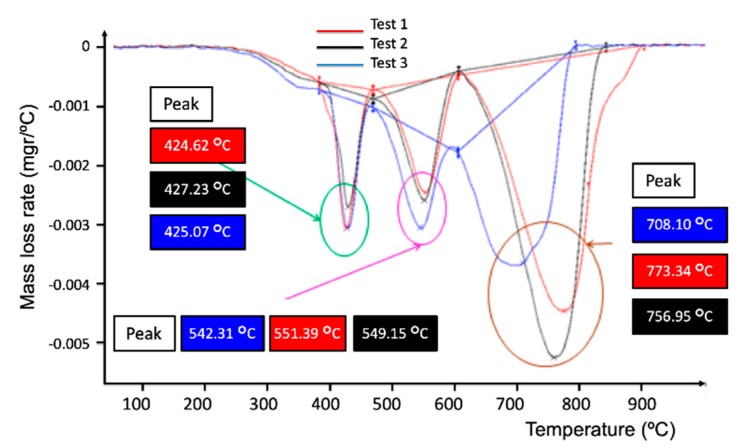
Derivative thermogravimetric (DTG) for semi-cured composite samples (Sample 2).

**Figure 5:**
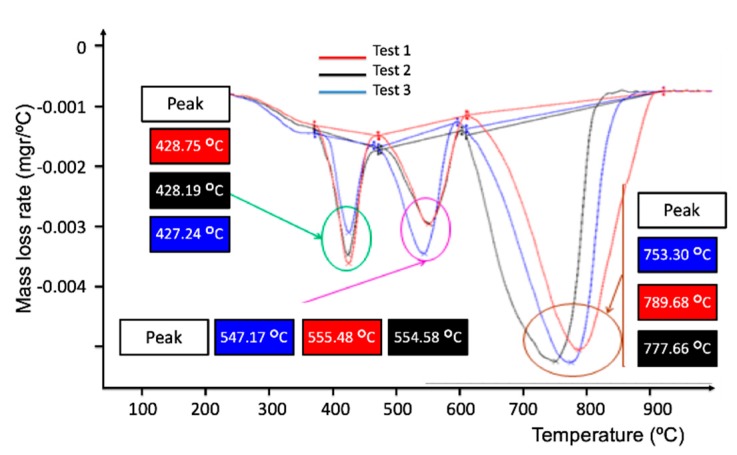
DTG for cured composite samples (Sample 3).

Derivative thermogravimetric (DTG) is a type of thermal analysis in which the rate of material weight changes upon heating is plotted against temperature. It is used to simplify reading the weight versus temperature thermogram peaks, which occur close together. The results of the DTG analysis for the semi-cured (Sample 2) and cured (Sample 3) samples are shown in Figure 4 and Figure 5, respectively. 

In both figures, three peaks corresponding to three phases were observed by the dynamic thermogravimetric studies in oxidative conditions, as specified in Table 1.

It can be noticed that in Figure 4, the first phase starts at about 425 °C, the second phase reaches a maximum rate near 550 °C, and finally, the third phase is near 790 °C. The third phase corresponds with the degradation of the residue. For both samples 2 and 3, the initial (lost) masses are shown in Table 2 and the mass losses are presented in Table 3. 

Table 4 displays the fraction percentages for Samples 2 and 3, after applying the cure phases from Equations (1)–(4).

**Figure 6:**
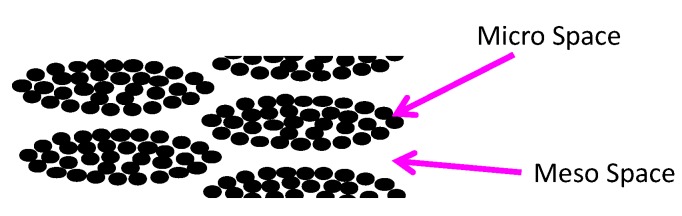
Micro and meso spaces between and inside tows.

The analysis of the decomposition process of the composites suggests that the early degradation step is due to the removal of the most volatile components and low molecular mass species, with a rate of about 35% for the three replicas of both samples. The second stage of the thermal decomposition would correspond to the scission of the polymer network, and the later decomposition stage would be related to the thermal degradation of the carbon fibers [11]. Table 5 displays the average and standard deviation values for the four studied fractions. The high standard deviations found for fractions P2 and P3 indicate a non-uniform curing process. This behavior is in good agreement with the composite internal double scale, as represented in Figure 6. The gap between fibers inside and between tows are around 5 μm and 500 μm respectively, and then the cure kinetic is different. 

**Figure 7:**
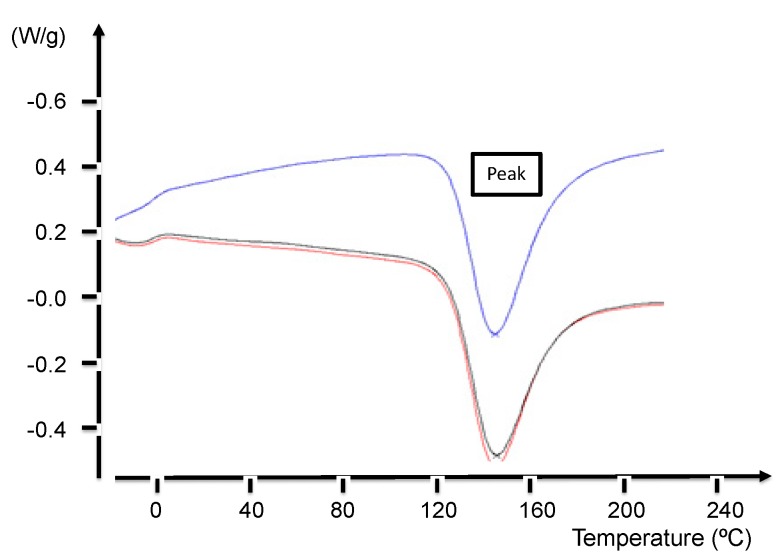
DSC measurements for Sample 1: exothermic heat curves at a rate of 10 °C/min.

Differential scanning calorimetry (DSC) was used to characterize the degree of cure of the epoxy carbon composites. Figure 7 shows the exothermic heat curves of the epoxy system for Sample 1 at a heating rate of 10 °C/min. The area under the curve, which is calculated from the extrapolated baseline at the end of the reaction, was used to assess the total heat of the cure, giving a value of HT = 135 J/g. In general, the curing reaction of a thermosetting resin can spread over a wide temperature range. However, considering the sensitivity and response limitation to heat changes, the isothermal DSC analysis is usually run in a moderate curing range of temperature [12]. According to the dynamic curing DSC curves displayed in Figure 6, the drop on the weight is a Ti = 125 °C and it reaches a minimum at Tpeak = 145 °C. This means that the temperature for optimum curing is in the range 125–145 °C.

**Figure 8:**
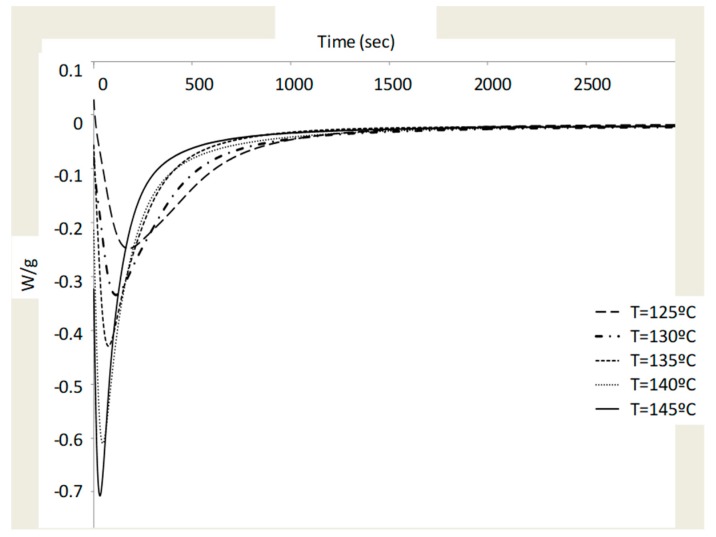
Isothermal DSC curves for composite samples.

The isothermal degree of cure was obtained from isothermal DSC curves (Figure 8). As expected, when the curing temperature increased, the time required to reach the minimum value became shorter. The isothermal heat of cure, ΔHi, was derived from the total area under the curve. The residual heat of reaction (ΔHR) is obtained by a cooling and heating process for each T_iso_ value. The values of ΔHi and ΔHR are shown in Table 6 at the T_iso_ temperature.

Then, the degree of cure at T_iso_ can be calculated through two equivalent expressions:

(9) $$\alpha_{\mathrm{Hi}}=\frac{\Delta H_{i}}{\Delta H_{T}}$$

(10) $$\alpha_{Hi}=\frac{\Delta H_{i}}{\Delta H_{T}}.$$

Table 7 shows the results obtained for the degree of cure, α, for each. As can be seen, there is no clear trend on the behavior of reaction heats, which suggests that a non-uniform process is occurring. This circumstance has also been observed in the TGA experiments, so it is interpreted that the information of both results is complementary and can help the interpretation of physical phenomena to be modeled with the cure equations.

### 3.2. Raman Spectroscopy

Raman scattering measurements of the three samples (1, 2, and 3) were performed under the same conditions: two accumulations of 120 s of exposure time and a neutral 0.6 density filter. The use of an attenuator has been revealed to be necessary to avoid the radiation damage on the composite samples. The Raman spectrum shown for each sample is obtained by calculating the average of the three points of one fiber after checking that several fibers gave similar results. In order to obtain the optimal conditions to perform the measurements, different combinations of exposure time and attenuators have been studied. Once the tests are finished, no significant changes are observed in the surface of the samples measured by Raman spectroscopy.

**Figure 9:**
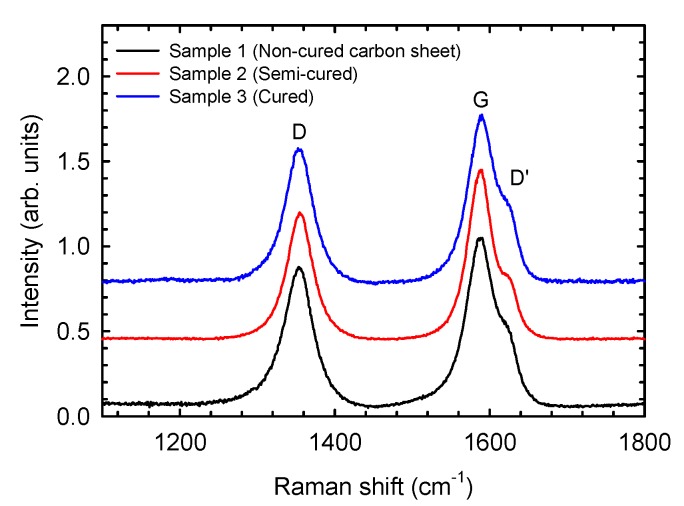
Raman spectra of three analyzed composite samples as a function of their degree of cure. The Raman spectra have been vertically shifted to improve the comparison among them.

Figure 9 shows the Raman spectra for the three composite fiber samples. The Raman spectra have been represented vertically to clarify their visualization. All samples have a peak located around 1352, 1585 and 1620 cm^−1^. The peak located at 1585 cm^−1^ is a consequence of ordered or graphitic carbon (known as G band), while the peaks located at 1352 cm^−1^ (D band) and 1620 cm^−1^ (D’ band) are assigned to disordered carbon atoms, which is usually explained by the double-resonance Raman mechanism in carbon [12,13]. With the present spectral resolution of our Raman spectrometer (below 3 cm^−1^), the D’ band appears as a shoulder of the G band and cannot be separated. Therefore, only the values of the D and G bands were used in our calculations.

The ratio between the intensities of both D and G bands gives us an idea of the crystalline order of the sample. The crystalline size, L_a_, is derived from the Knight formula [14,15],

(11) $$L_{a}\left(\mathrm{nm}\right)=(2.4\times 10^{-10})\;\lambda^{4}{\left(\frac{I_{D}}{I_{G}}\right)}^{-1}$$

where λ is the laser line wavelength in nanometer units, and I_D_ and I_G_ are the intensities of the D and G bands, respectively [16,17]. The values for the crystalline order and crystallite sizes for the composite samples with different cure degrees are displayed in Table 8.

The results of Raman spectroscopy indicate that the crystalline order increases in the cured samples, while the crystallite size decreases with the cure. These results are consistent with the results obtained with DTG studies. As can be seen, the degree of cure is directly related to an increase in temperature peaks, as shown in DTG studies, which results in a rise of the crystalline order.

## 4. Conclusions

Thermal analysis and Raman spectroscopy technologies provide relevant information for the modeling of the kinetic behaviour of the resins in composite materials. The control of the degree of curing of composite parts is fundamental for the optimization of the mechanical properties. The degree of polymerization of carbon–epoxy composites was investigated through several techniques such as FESEM, TGA, DSC, and Raman spectroscopy. FESEM micrographs reveal that the surface of the carbon fibers is better defined as the cure time becomes longer, due to the homogeneous redistribution of covering resins. TGA and DSC experiments confirm that the thermal characteristics of cured samples depend on the applied cure process. Raman spectroscopy offers an assessment of the crystalline order and crystallite sizes through the ratio between the intensities of D and G bands, which corresponds to the disordered and ordered phases of graphitic carbon, respectively. The results show that both Raman spectroscopy and thermal analysis are useful and complementary techniques for evaluating the degree of cure in composite materials. The technologies studied in this article are easily extrapolated to other materials or different applications, such as biomaterial for tissue engineering or biocomposites. Nowadays, many biotissues are manufactured with thermoset resins that need proper curing to achieve maximum mechanical properties. The problems associated with correct curing such as shrinkage or surface defects can be easily improved with these techniques.

## Figures and Tables

**Table 1:** Thermal degradation temperature. Samples 2 and 3.

Sample Number	Tpeak1	Tpeak2	Tpeak3
Sample 2	427 °C	551 °C	773 °C
Sample 3	430 °C	553 °C	790 °C

**Table 2:** Initial mass for Samples 2 and 3.

Scan	P_0_ (mg) Sample 2	P_0_ (mg) Sample 3
C1	1.1655	1.1276
C2	1.1630	1.1634
C3	1.0930	1.1214

**Table 3:** Mass loss in the tests of Samples 2 and 3.

Sample 2			Sample 3		
Tc = 427 °C	Tc = 551 °C	Tc = 773 °C	Tc = 430 °C	Tc = 553 °C	Tc = 790 °C
Mass Loss P_1_ (mg)	Mass Loss P_2_ (mg)	Mass Loss P_3_ (mg)	Mass Loss P_1_ (mg)	Mass Loss P_2_ (mg)	Mass Loss P_3_ (mg)
0.4103	0.0681	0.6859	0.4098	0.2497	0.4648
0.4192	0.2413	0.5009	0.4174	0.5555	0.1862
0.3805	0.0359	0.6755	0.3855	0.0276	0.7073

**Table 4:** Fraction percentages for Samples 2 and 3.

Fraction	Sample 2			Sample 3		
	Rep. 1 (%)	Rep. 2 (%)	Rep. 3 (%)	Rep. 1 (%)	Rep. 2 (%)	Rep. 3 (%)
P1	35	36	34	36	35	34
P2	5	20	3	22	47	2
P3	58	43	61	41	16	63
Residue	2	1	2	1	2	1

**Table 5:** Standard deviation and average for Samples 2 and 3.

Fraction	Sample 2		Sample 3	
	Average	Standard Deviation	Average	Standard Deviation
P1	35.0	1.0	35.0	1.0
P2	9.3	9.3	23.7	22.5
P3	54.0	9.6	40.0	23.5
Residue	0.7	0.6	1.3	0.6

**Table 6:** ΔH_i_ and ΔH_R_ for five isothermal experiments.

T_iso_ (°C)	ΔH_i_ (J/g)	ΔH_R_ (J/g)
125	132.70	2.87
130	134.50	3.12
135	118.26	3.50
140	135.99	3.62
145	123.60	6.12

**Table 7:** Values of DOC for Sample 2.

T_iso_ (°C)	α_HR_	α_Hi_
125	0.98	0.98
130	0.98	0.99
135	0.97	0.88
140	0.97	1.00
145	0.95	0.92

**Table 8:** Crystalline order and crystallite size derived from the Knight formula.

Sample	I_D_ (a.u.)	I_G_ (a.u.)	I_D_/I_G_	L_a_ (nm)
1 (Semi-cured)	0.73	0.98	0.74	25.8
2 (Cured)	0.79	0.98	0.81	23.9
3 (Carbon sheet)	0.81	0.97	0.84	23.0
